# Cas rare de sangsue laryngée chez un homme de 70 ans

**DOI:** 10.11604/pamj.2017.26.19.11412

**Published:** 2017-01-16

**Authors:** Said Anajar, Mohammed Tatari, Jawad Hassnaoui, Reda Abada, Sami Rouadi, Mohammed Roubal, Mohammed Mahtar

**Affiliations:** 1 Service ORL et Chirurgie Cervico-faciale, Hôpital 20 Août, Casablanca, Maroc

**Keywords:** Dyspnea, hemoptysis, Leech, Leech, dyspnea, haemoptysis

## Abstract

La dyspnée laryngée représente une urgence extrême en otorhinolaryngologies. Elle répond à des étiologies multiples et reste souvent graves. La dyspnée laryngée causée par une sangsue est exceptionnellement décrite dans la littérature surtout chez l’adulte. Nous rapportons une observation particulière d’infestation de l’arbre respiratoire par une sangsue ayant entraîné une dyspnée laryngée.

## Abstract (en)

Foreign bodies in the upper respiratory tract are one of the most difficult otolaryngological emergencies; leeches are a very rare foreign bodies in the world. We report the case of a 70-year-old man with no past medical history presenting with laryngeal dyspnea associated with low abundant paroxysmal hemoptysis. The patient underwent nasofibroscopy showing the presence of a living and mobile organism at the subglottic level evoking a leech. Extraction was carried out under local anesthesia using laryngoscope and Magill forceps. The presence of a leech as a foreign body in the upper respiratory tract should be suspected in patients with a recent history of consumption of non-potable water.

## Introduction

La dyspnée laryngée représente une urgence extrême en otorhinolaryngologies. Elle répond à des étiologies multiples et reste souvent graves. La dyspnée laryngée causée par une sangsue est exceptionnellement décrite dans la littérature surtout chez l’adulte. Nous rapportons une observation particulière d’infestation de l’arbre respiratoire par une sangsue ayant entraîné une dyspnée laryngée.

## Patient et observation

**Figure 1:**
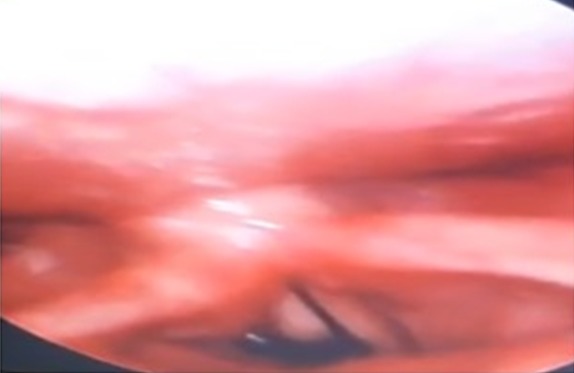
Nasofibroscopie montrant une sangsue au niveau glottique

**Figure 2:**
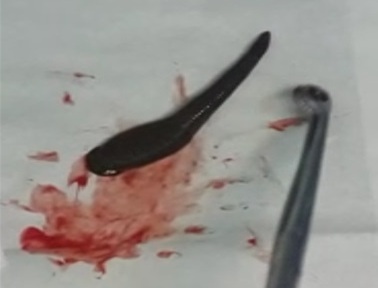
Sangsue après extraction

Il s’agit d’un patient âgé de 70 ans, sans antécédent pathologique particulier, admis pour une dyspnée laryngée et hémoptysie de faible abondance. Les symptômes ont été: une sensation de corps étranger augmentant progressivement de volume avec démangeaisons pharyngées avec une gêne respiratoire. L’examen clinique à l’admission trouve un patient stable sur le plan hémodynamique, polypnéique à 28 cycles par minute avec une saturation en oxygène à 94% à l’air ambiant, un tirage sus sternale, avec une pâleur cutanéo conjonctivale. Le reste de l’examen somatique était normal. La numération de la formule sanguine montre une anémie hypochrome microcytaire à 7 g/dl d’hémoglobine. Le reste du bilan biologique était normal notamment l’ionogramme sanguin et le bilan de l’hémostase. La sangsue a été retrouvée après réalisation d’une nasofibroscopie, elle était fixée dans l’étage sus glottique obstruant la totalité de la lumière laryngée (Figure 1). L’extraction a été réalisée sous anesthésie locale (xylocaine 5% spray) à l’aide d’un laryngoscope et d’une pince de Magill (Figure 2). L’évolution est immédiatement favorable avec une régression des signes respiratoires. L’interrogatoire révèle que le patient provient d’une région rurale buvant exclusivement de l’eau de puits non traitée.

## Discussion

Les sangsues des voies aérodigestives supérieurs ne sont pas rares au Maroc. L’infestation à été principalement décrite dans les pays méditerranéens, en Afrique et en Asie [1]. La sangsue est un ver d’eau douce qui peut se retrouver accidentellement dans les voies aériennes de l’homme après consommation d’eau de source ou de puits naturels ou après des baignades en eaux stagnantes (lacs, barrages). En effet, la salive du parasite secrète une substance anticoagulante et anesthésique (hirudine) responsable des manifestations hémorragiques. Un état de choc est possible en cas d’anémie sévère [2]. Plusieurs localisations ont été décrites chez l’adulte et l’enfant. Les plus fréquentes sont la localisation nasale à l’origine d’obstruction et d’épistaxis récidivantes [3]. L’infestation pharyngée ou laryngotrachéale peut associer une dyspnée obstructive, parfois asphyxiante au syndrome hémorragique [4, 5]. Chez notre patient la localisation été sus glottique associant hémoptysie et dyspnée laryngée. La sangsue ingère le sang dès sa fixation, les quantités peuvent être considérables et peut atteindre progressivement près de dix fois son poids [6], ce qui peut provoquer une anémie sévère [7].

La sangsue secrète l’hirudine, puissant inhibiteur de la thrombine, et d’autres facteurs anticoagulants comme le facteur plasminogene activating factor (IXa). Sa salive contient également des enzymes comme l’antiélastase, l’antiplasmine, l’antitrypsine [8, 9]. Ces propriétés anticoagulantes, anti-inflammatoires et vasodilatatrices justifient son utilisation dans les greffes de membres, les hématomes et les thrombophlébites [10]. La symptomatologie clinique est variable selon la localisation dans les voies aérodigestives. Il s’agit d’épistaxis récidivantes, d’obstruction nasale, ou de sensation de corps étranger mobile pour les localisations nasales ou nasopharyngées. Le diagnostic différentiel est celui des tumeurs bénignes ou malignes du nasopharynx. La localisation oropharyngée occasionne des crachats sanglants et/ou une dysphagie haute. L’atteinte laryngée est moins fréquente. Elle peut être responsable d’une symptomatologie bruyante. L’évolution peut être rapidement mortelle avec des hémoptysies, un changement de la voix, une toux sèche et une détresse respiratoire. Le tableau clinique peut être trompeur, révélé uniquement par une anémie insolite, le plus souvent hypochrome microcytaire. Quel que soit son site d’implantation, le retrait du parasite est difficile. Il peut se faire sous anesthésie locale ou générale pour les localisations laryngées et certains auteurs préconisent l’application d’adrénaline, de cocaïne, de solution saline ou de vinaigre [7]. La manipulation de la région laryngée sous anesthésie locale est inconfortable pour le patient. L’intubation trachéale pour l’anesthésie générale peut se compliquer d’une rupture de la sangsue ou de son inhalation, entraînant la persistance du saignement [6]. Il est préférable de procéder à une anesthésie générale par induction, sans intubation trachéale. En effet, l’absorption de produit anesthésique par la sangsue peut entraîner sa paralysie, facilitant son ablation [6]. Chez notre patient l’extraction a été réalisée sous anesthésie locale.

## Conclusion

Les sangsues devraient être soupçonnées comme un corps étranger de voies respiratoires chez les patients ayant une histoire récente de consommation d'eau non traité, il doit être diagnostiqué et traité rapidement. La nasofibroscopie permet un diagnostic précis pour détecter une sangsue dans le larynx. La prévention reste le meilleur traitement basée simplement sur des mesures d'hygiène comme ne pas boire d’eau non traité et utiliser des filtrer d’eau.
